# Improved accuracy of digital implant impressions with newly designed scan bodies: an in vivo evaluation in beagle dogs

**DOI:** 10.1186/s12903-021-01986-2

**Published:** 2021-12-07

**Authors:** Ruoxuan Huang, Yuanxiang Liu, Baoxin Huang, Fengxing Zhou, Zhuofan Chen, Zhipeng Li

**Affiliations:** 1 Hospital of Stomatology, Guanghua School of Stomatology, Sun Yat-Sen University, Guangdong Provincial Key Laboratory of Stomatology, No. 56, Lingyuan west road, Guangzhou, 510060 China; 2 KTJ Dental Group, Shenzhen, China

**Keywords:** Dental implant, Digital impression, Scan body, Edentulous, Accuracy

## Abstract

**Background:**

The accuracy of digital impressions for fully edentulous cases is currently insufficient for routinely clinical application. To overcome the challenge, a modified scan body was introduced, which demonstrated satisfactory accuracy in vitro. The aim of this study was to evaluate the accuracy of digital impressions using the modified scan bodies with extensional structure versus scan bodies without extensional structure in mandible with two implants in beagle dogs.

**Methods:**

The unilateral mandibular second premolar to second molar were extracted in four beagle dogs. Twelve weeks later, two implants were placed. Five repeated digital impressions were performed with an intraoral scanner on each dog using each of the two different scan bodies: Group I—scan body without extensional structure (SB); Group II—scan body with extensional structure (SBE). The scans were exported to Standard Tessellation Language (STL) files to serve as test data. The dogs were sacrificed and the dissected mandibles were digitalized with a lab scanner to provide reference data. Linear and angular deviations were calculated in an inspection software for accuracy assessment. Statistical analysis was performed with two-way ANOVA. The level of significance was set at α = 0.05.

**Results:**

For trueness assessment, the mean of absolute linear/angular deviations were 119.53 μm/0.75 degrees in Group I and 68.89 μm/0.36 degrees in Group II. SBE was more accurate than SB regarding both linear (*p* = 0.008) and angular (*p* = 0.049) deviations. For precision assessment, the mean of absolute linear/angular deviations were 63.01 μm/0.47 degrees in Group I and 38.38 μm/0.24 degrees in Group II. No significant difference was found.

**Conclusions:**

The application of SBE significantly improved the trueness of digital impressions in mandible with two implants compared to SB. No significant difference was found in terms of precision.

## Background

Digital impressions using intraoral scanners have been more and more popular in dental implant treatment. Compared to conventional impression technique, digital impressions eliminate several procedures such as dispensing and setting of impression materials, disinfection, and stone cast pouring and shipping. The simplified workflows not only improve time efficiency, but also reduce the chances of deformation [1–3]. The virtual models can be digitally transferred and stored, which facilitates communication, reduces costs and saves space [4, 5]. Additionally, digital impressions have been reported to perform better regarding patients’ acceptance [6].

Digital impressions have been recommended for single-unit or short-span implant rehabilitations [7, 8]. For complete-arch implant rehabilitation, however, the widespread application of digital impressions remains controversial because the scanning accuracy and influencing factors haven’t been well demonstrated [7]. As defined in previous studies, accuracy consists of trueness and precision [2, 9, 10]. Trueness is defined as closeness of the test scans to the reference scans, and precision is defined as closeness of the repeated scans to each other [2, 9, 10]. Several laboratory-based studies have been conducted and most of the results indicated that digital impressions for complete-arch implant rehabilitation exhibited superior or equal accuracy compared to conventional impressions [10–13]. A study assessing 8 different intraoral scanners suggested that the errors produced by the scanners in complete-arch digital implant impression ranged from 31 to 344 μm, among which True Definition and Trios 3 showed significantly higher accuracy [14]. The extraoral application of the scanners excluded the effect of limited space, unstable mucosa, reflective saliva and blood in the oral cavity, which could negatively influence the scanning accuracy [15].

The number of clinical trials on the accuracy of digital implant impressions was quite limited [16–20]. Randomized controlled trials (RCT) reported that digital impressions were as accurate as conventional impressions and could provide clinically satisfactory outcomes [16, 18, 20]. An in vivo study by Andriessen et al., however, concluded that the accuracy of digital impressions was not acceptable for clinical application [19]. The major limitation of the clinical trials was that the accuracy was usually assessed by fit of the framework or clinical outcomes, which were mostly based on subjective clinical experience, instead of numeric values due to the lack of reference data representing the ‘true’ position of dental implants in the oral cavity [15, 21]. Considering the aforementioned drawbacks of in vitro study and clinical trial, an animal experiment may be a preferable alternative. It can mimic a clinical situation and, at the same time, provide reference data. However, there has been a lack of animal experiment on digital implant impressions.

As stated in multiple studies, major challenge for scanning fully edentulous arch lies in smooth mucosa surface without stable morphological characteristics, leading to accumulated stitching errors [15, 22]. Besides, the uniform scan bodies make it difficult for the scanning devices to distinguish one from another. A recent systematic review suggested that using splinted scan body could be helpful to overcome the difficulties [15]. A few innovative techniques have been introduced and tested [22–24]. In an in vitro study, modified scan bodies were produced and investigated [25]. The scan body was designed with a rigid bar extended from the cylindrical segment, which could provide stable reference points for stitching process at the inter-implant regions. The study has demonstrated that the scan bodies with extensional structure could improve scanning accuracy compared to scan bodies without extensional structure in vitro [25]. The scanning accuracy of the scan bodies in vivo needs to be further investigated.

In this study, long-span edentulous ridge was created in the mandible of beagle dogs to mimic the clinical situation of fully edentulous arch. The purpose of this study was to evaluate the accuracy of digital impressions in mandible with two implants using newly designed scan bodies with extensional structure versus scan bodies without extensional structure in beagle dogs. The null hypothesis was that digital impressions using scan bodies with or without extensional structure exhibited similar accuracy.

## Methods

### Animals

Four male beagle dogs aged 17–20 months and weighing 12–15 kg (Guangdong national beagles resources research center, Guangdong, China) were included in this study. All animals exhibited a fully erupted permanent dentition. The animals were housed in separate cages at Laboratory Animal Center of the University. The animals received a soft-food diet twice daily and had ad libitum access to water during the experimental period. The experiments started after a 2-week adaptation period. The study protocol was approved by the Institutional Animal Care and Use Committee (IACUC) of Sun Yat-Sen University (SYSU-IACUC-2018-000248), and all the procedures were carried out in accordance with the IACUC of Sun Yat-Sen University. The study was reported in accordance with the ARRIVE guidelines.

### Surgical procedures

Two surgical stages were involved in this study and were both carried out under general anesthesia. The anesthesia was induced by Xylazine Hydrochloride (0.05 ml/kg/i.m., Sumianxin, Jilin, China) and maintained with pentobarbital sodium (30 mg/kg/i.v., Sigma, Saint Louis, Missouri, USA). Mepivacaine Hydrochloride (Mepivacaine 36 mg, Adrenaline 0.018 mg, Septodont, France) was applied for local anesthesia. Penicillin G (300,000 i.u., Pen-B®, Pfizer Inc., Lee’s Summit, USA) was administrated during and after each surgical stage every 48 h for 10 days.

During surgical stage 1, the unilateral mandibular second, third and fourth premolar (P2, P3, P4), and the first and second molar (M1, M2) were carefully sectioned and extracted. Surgical stage 2 was conducted 12 weeks after tooth extraction. Following flap elevation and osteotomies, two bone level implants (Astra Tech Implant System, Dentsply Sirona, Pennsylvania, USA) with a diameter of 3.5 mm and a length of 8 mm were placed in parallel at the edentulous region via freehand surgery. The inter-implant distance was approximately 18 mm. Cover screws were connected. The flaps were closed using resorbable sutures, and submerged healing was allowed for 12 weeks.

### Intraoral scanning

**Fig. 1:**
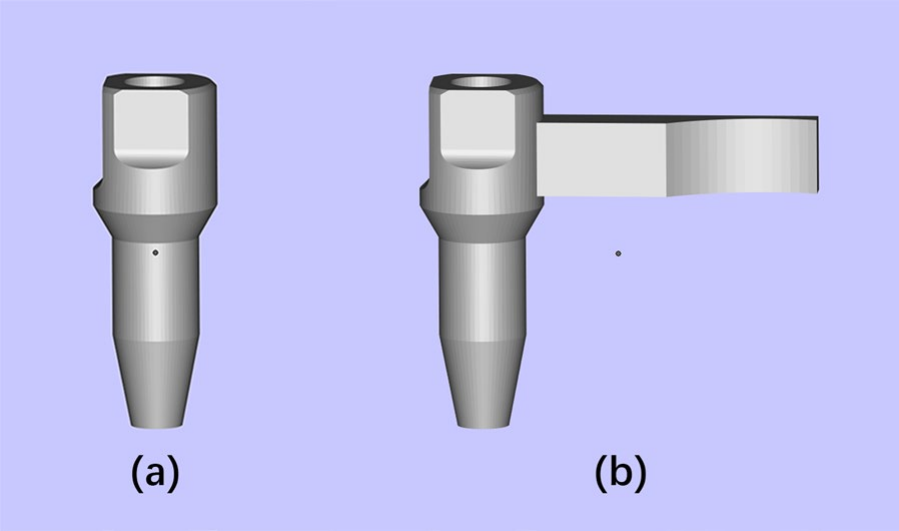
CAD files of the scan bodies. **a** Scan body without extensional structure. **b** Scan body with extensional structure

Twelve weeks after implants placement, intraoral scanning was performed under general and local anesthesia as described above. Mucoperiosteal flaps were elevated to expose the platform of the implants. Healing abutments were connected to the implants, and the flaps were sutured subsequently. Two different scan bodies were used in this study (Fig. 1). The scan bodies were fabricated with grade 5 titanium alloy (Gialloy Ti-5, SRL Dental GmbH, Ludwigshafen, Germany) sandblasted with 100 μm alumina powder at 0.5 MPa, and were subjected to autoclave sterilization.

Group I (control): scan body without extensional structure (SB). The scan body was 5 mm in diameter and 14 mm in height.

Group II (test): scan body with extensional structure (SBE). The scan body was a one-piece unit. The cylindrical segment of SBE was the same as SB, and the extensional structure was 11 mm in length.

**Fig. 2:**
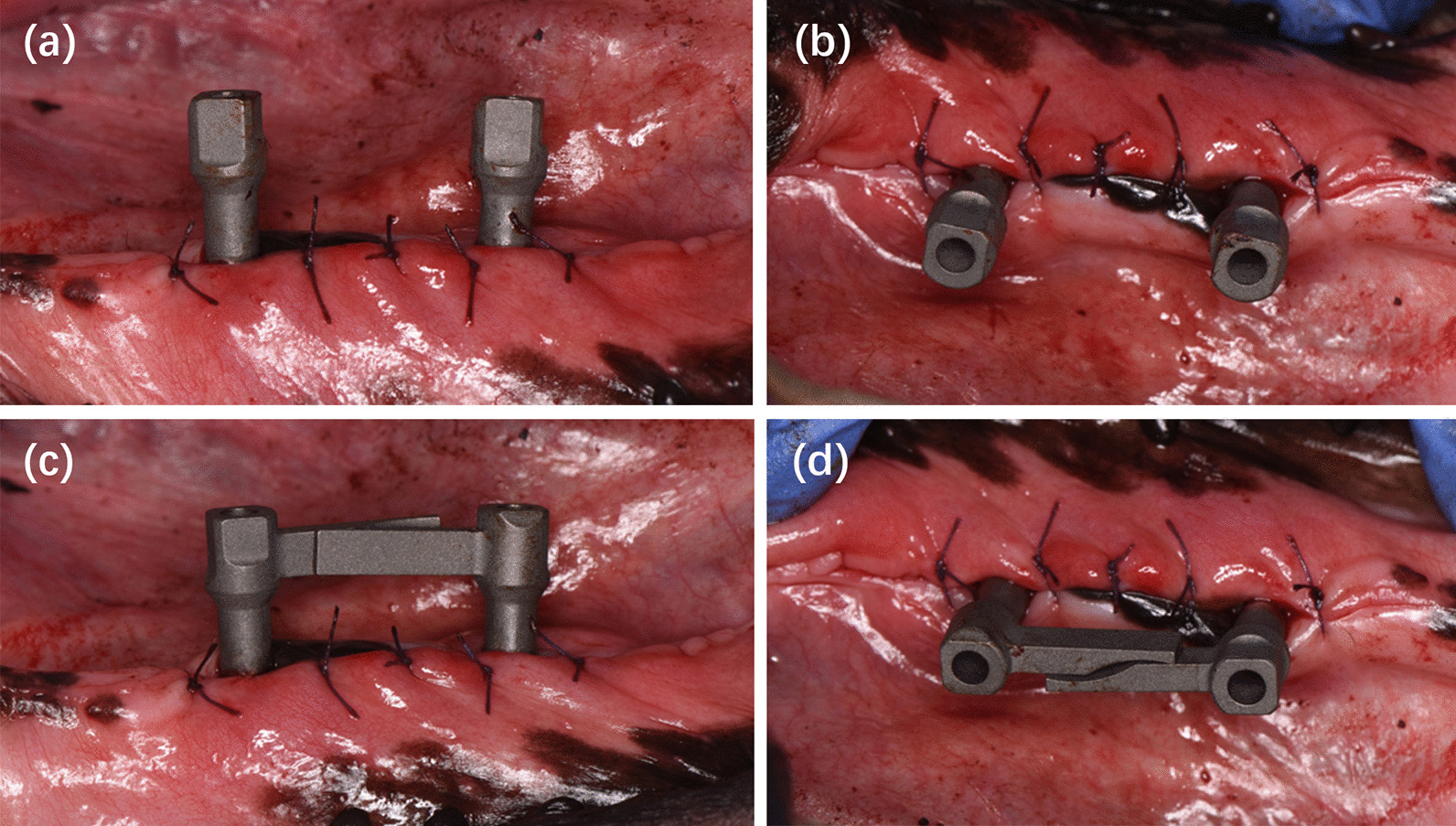
Intraoral scanning was performed with two different scan bodies connected to the implant. **a**, **b** Group I using SB. **c**, **d** Group II using SBE

**Fig. 3:**
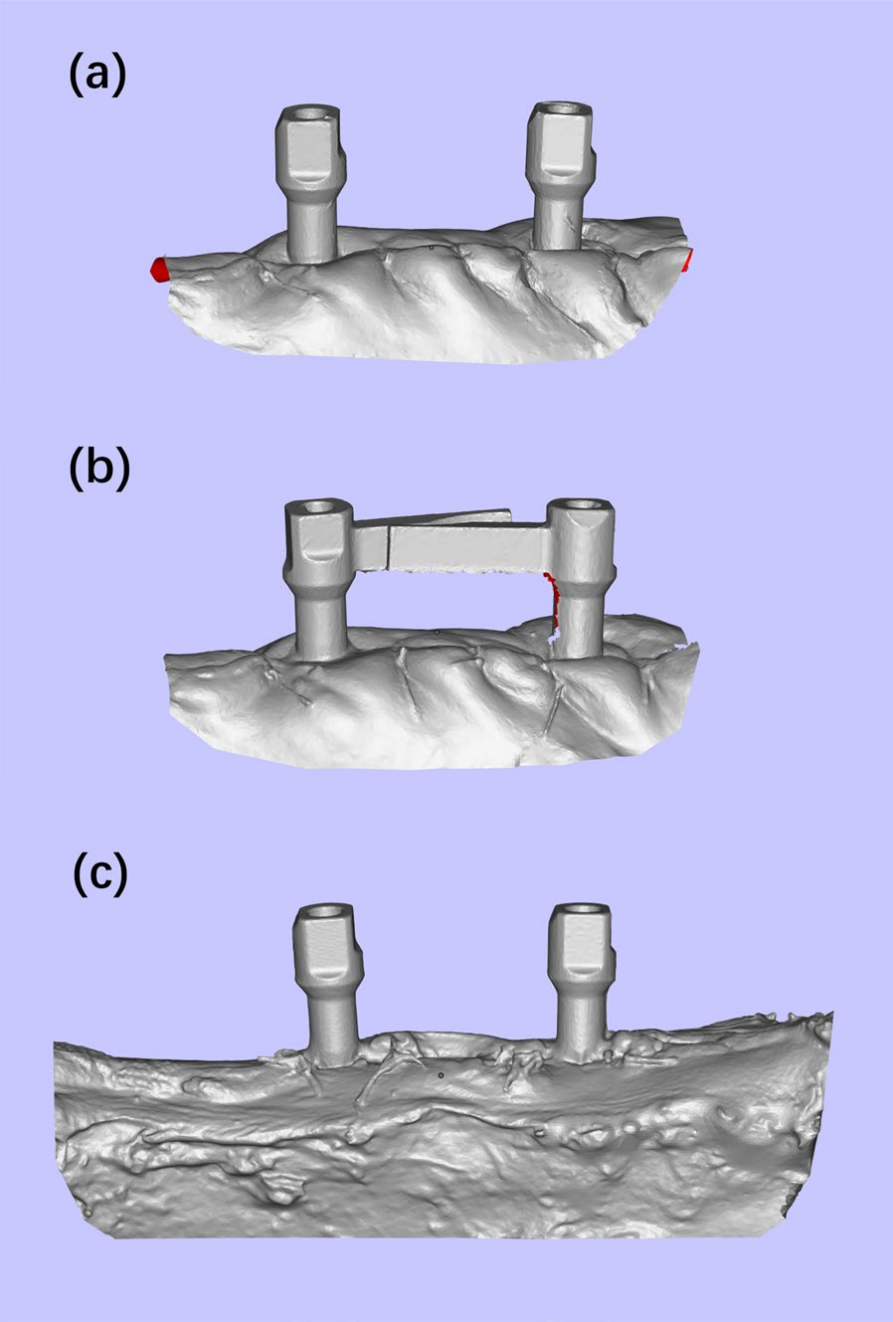
The test scans obtained with an intraoral scanner and the reference scans obtained with a lab scanner. **a** Test scan in Group I. **b** Test scan in Group II. **c** Reference scan

The scan bodies were connected to the implants and tightened to approximately 10 Ncm using a manual torque wrench (Fig. 2). In SBE group, the scan bodies were positioned on the implants and rotated, making the extensional bars in contact with each other, before tightened up. All the scanning was performed by an experienced operator. For each group, five repeated scans were made using an intraoral scanner (TRIOS3, 3Shape, Copenhagen, Denmark). The scanning was started from the lingo-occlusal surface with the scanner tip moving from distal scan body to mesial scan body. The buccal surface was captured subsequently. No tooth was captured during the scanning procedure so as to mimic a fully edentulous arch. Totally 10 scans for each dog were produced and exported to open-format Standard Tessellation Language (STL) files to serve as test scans (Fig. 3a, b).

### Reference datasets

The animals were sacrificed with overdose pentobarbital sodium (120 mg/kg/i.v.). The mandibles were dissected. The scan bodies without extensional structure were connected to the implants and were scanned using a laboratory scanner (D2000, 3Shape, Copenhagen, Denmark) with a reported precision of 5 μm. The 3D images were exported to open-format STL files to serve as reference scans (Fig. 3c).

### Data analysis

**Fig. 4:**
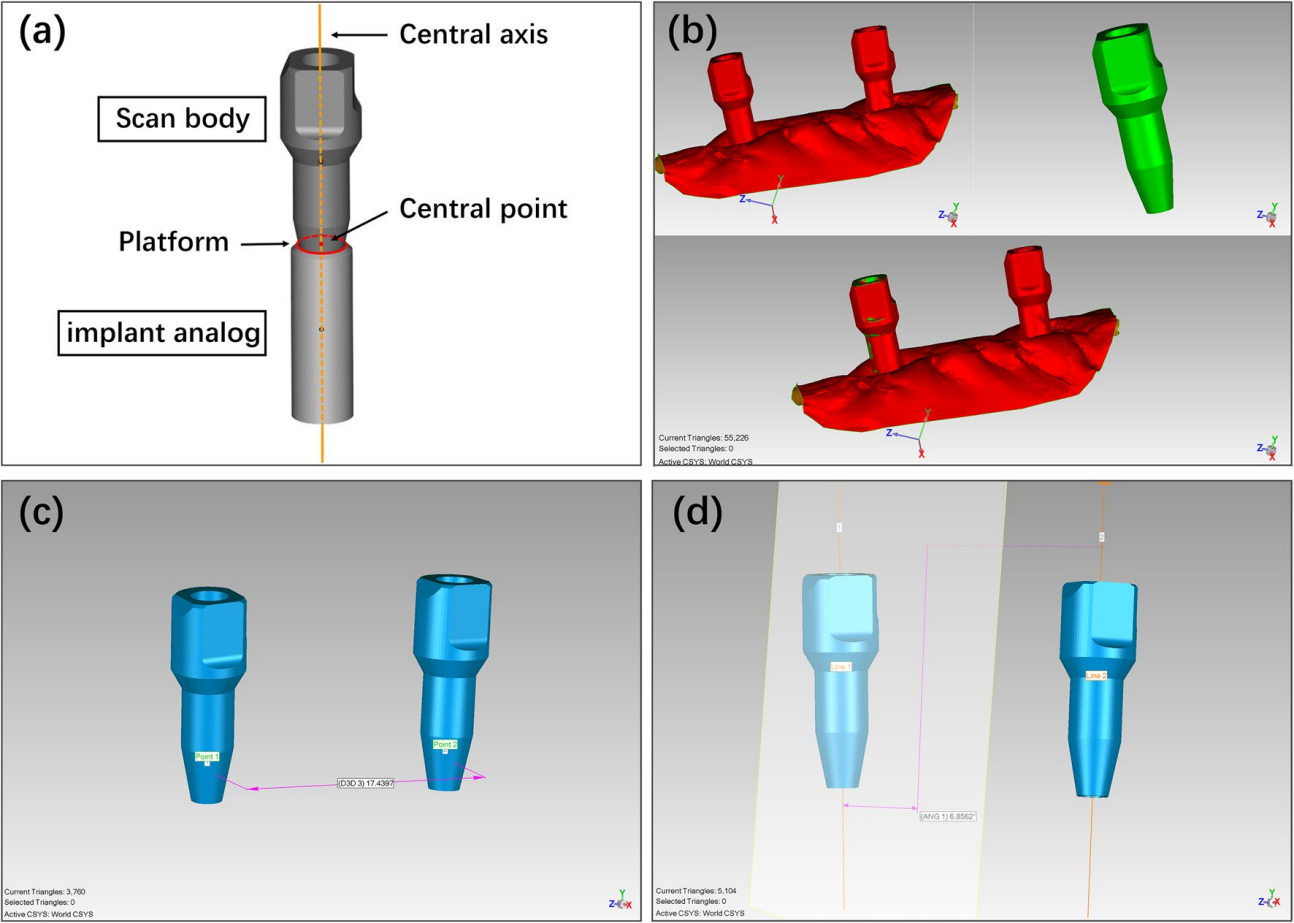
Data analysis process. **a** Central point at the level of implant platform and longitudinal central axis were created on the CAD files of SB. **b** Alignment of the CAD file of SB (green) and the digitalized scan bodies in the scan (red). **c** Inter-implant distance was measured by the distance between the two central points. **d** Inter-implant angle was measured by the angle between the two central axes

Deviation of inter-implant distance (linear deviation) and inter-implant angle (angular deviation) was used for accuracy assessment. Central point at the level of implant platform and longitudinal central axis were created on the CAD file of SB (Fig. 4a). The CAD file, together with the STL files of the test scans and reference scans, were imported to inspection software (Geomagic Control 2015, 3D systems, Rock Hill, SC, USA). The CAD file of SB was then aligned to the digitalized scan bodies in the test/reference scans using best-fit algorithm in both SB and SBE groups (Fig. 4b). Inter-implant distance (ID) was measured by the distance between the two central points, and inter-implant angle (IA) was measured by the angle between the two central axes (Fig. 4c, d).

Deviations of ID and IA between the test scans and the corresponding reference scan were calculated and recorded as ΔID and ΔIA, respectively. The absolute values of ΔID and ΔIA were used for trueness evaluation.

The mean of ΔID and ΔIA for each dog within each group were calculated. Comparisons between ΔID/ΔIA and the corresponding mean values were performed, the absolute values of which were used for precision evaluation.

### Statistics

Statistical analysis was performed using R version 3.6.2. Difference between groups in trueness and precision was evaluated using two-way ANOVA. The between-subject factor was the scan body. The within-subject factor was the dog. The level of significance was set at α = 0.05.

## Results

No implant was loosened in all the four dogs. No complication, such as peri-implantitis, was observed throughout the study. So, a total of four mandibles were used for assessment.

**Fig. 5:**
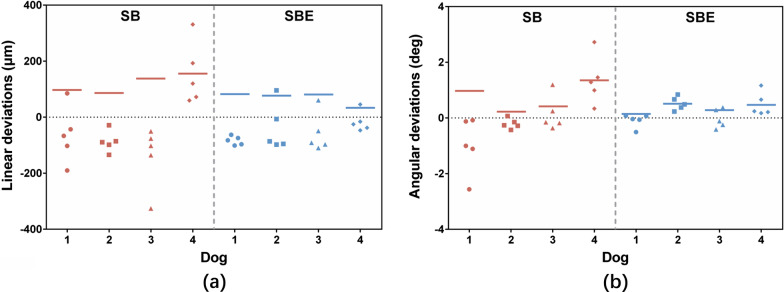
Linear and angular deviations of all the scans. **a** Linear deviations. **b** Angular deviations. The lines indicated mean of absolute values of the deviations for each dog within each group

Linear and angular deviations of all the scans were shown in Fig. 5. Positive values indicated that the test scans exhibited larger inter-implant distance/angle compared to the reference scans.

**Fig. 6:**
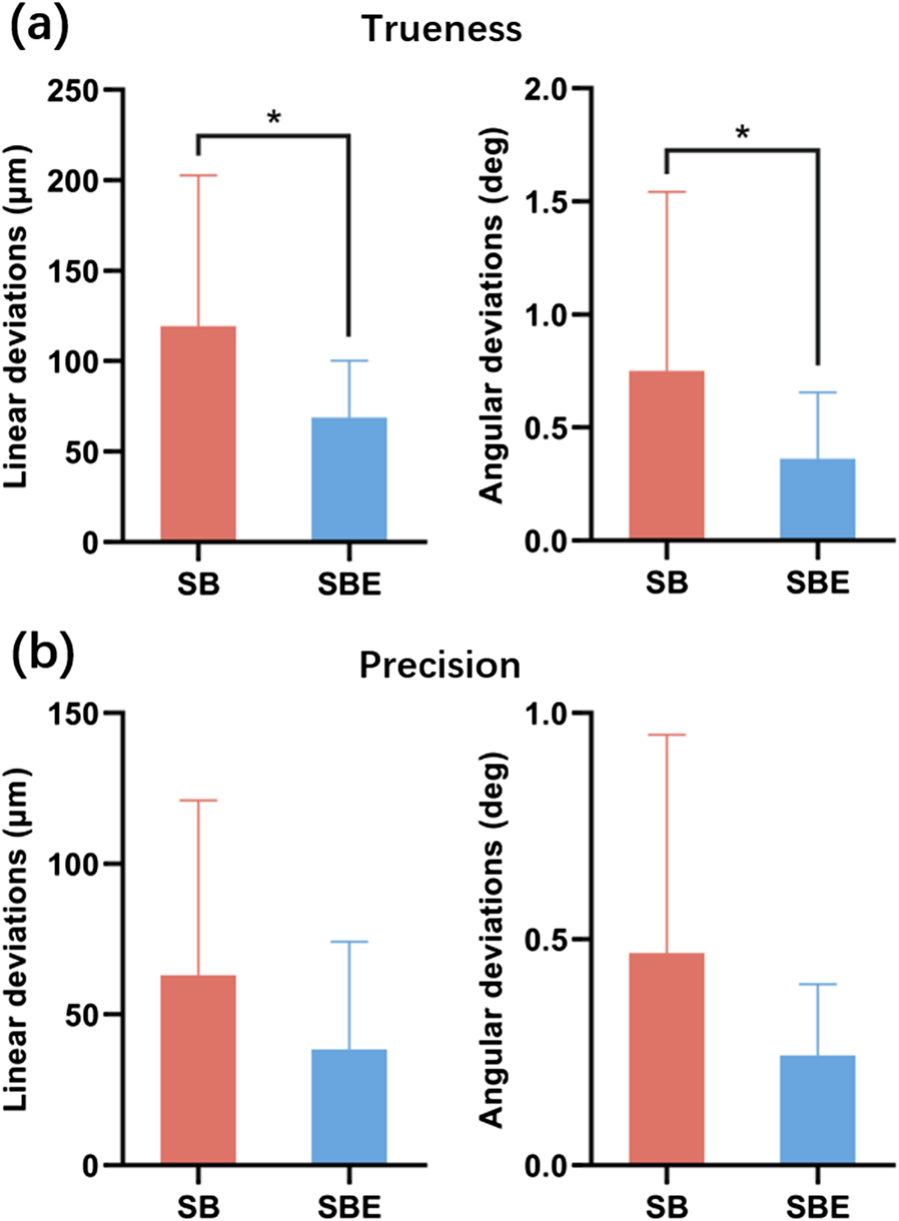
Results of trueness and precision evaluation. **a** Trueness. **b** Precision. *indicates statistical significance

In terms of trueness, the mean (SD) of absolute linear deviations were 119.53 (83.27) μm in Group I and 68.89 (31.34) μm in Group II. The mean (SD) of absolute angular deviations were 0.75 (0.79) degrees in Group I and 0.36 (0.29) degrees in Group II. SBE was more accurate than SB regarding both linear (*p* = 0.008) and angular (*p* = 0.049) deviations (Fig. 6a).

In terms of precision, the mean (SD) of absolute linear deviations were 63.01 (58.06) μm in Group I and 38.38 (35.80) μm in Group II. The mean (SD) of absolute angular deviations were 0.47 (0.48) degrees in Group I and 0.24 (0.16) degrees in Group II. No significant difference was found for both linear (*p* = 1.000) and angular (*p* = 1.000) deviations (Fig. 6b).

## Discussion

This study evaluated the accuracy of digital impressions on edentulous mandible with two implants using newly designed scan bodies with extensional structure versus scan bodies without extensional structure in beagle dogs. The null hypothesis was partially rejected. SBE exhibited better accuracy than SB in terms of trueness. To the authors’ knowledge, this is the first study that evaluated the accuracy of digital impressions by an animal experiment.

To our knowledge, there was only one in vivo study assessing the accuracy of digital impressions on edentulous mandibles with two implants [19]. The definite stone casts from conventional impressions were taken as references, and 100 μm and 0.4 degrees were defined as thresholds for clinical acceptable linear and angular deviations. The results presented a mean linear deviation of 226.0 μm and a mean angular deviation of 2.582 degrees, and only one out of the 25 scans exhibited acceptable deviations [19]. The mean linear and angular deviations in our study were much lower, which was partially owe to the development of the intraoral scanners. Considering the aforementioned threshold, only 11 out of 20 scans were clinically acceptable using SB in terms of linear deviations, while 18 out of 20 scans met the criteria using SBE. It is worth noting that linear deviations of the remaining two scans in SBE group were closed to 100 μm, while two scans in SB group exhibited high deviations of more than 300 μm. In terms of angular deviations, 11 and 13 scans were clinically acceptable using SB and SBE, respectively. To the authors’ knowledge, no consensus has been reached regarding acceptable levels of misfit. Jemt et al. stated that a discrepancy of 150 μm was clinical acceptable [26]. In this case, all the 20 scans in SBE group exhibited satisfactory accuracy. Additionally, it was interesting to find that most of the linear deviations were negative values, which implied that the virtual models obtained by intraoral scanning showed smaller inter-implant distances in comparison with the actual situation. The finding was in consistence with an in vitro study involving six or eight implants, which also found that the virtual models were ‘shrunk’ [27]. Further studies are needed to clarify the mechanism.

Multiple in vitro studies have been conducted to assessed the accuracy of digital impressions for fully edentulous arch, and a diversity of results has been reported [10–12, 14, 22, 28–32]. In an in vitro study, eight intraoral scanners were evaluated and showed mean deviations ranged from 31 to 344 μm, among which TRIOS exhibited a mean deviation of 32 μm [14]. Kim and coworkers, however, reported that the median of linear deviations of digital impressions using TRIOS was 177.4 μm, which was significantly higher than conventional impressions (72.2 μm) [28]. The high degree of heterogeneity of the results could be explained by differences in study design and methodology used for accuracy assessment. The present study was an in vivo study while the results were within the previously reported ranges. It could be speculated that the accuracy in this study might be compromised compared to an in vitro protocol because the complicated intraoral condition, might exert a negative impact on scanning accuracy [2]. What’s more, the intraoral scanning was performed immediately after suturing to mimic the clinical situation of impression taking immediately after implants placement or 2nd stage surgery. Higher accuracy could be anticipated with more stable mucosa and minor bleeding if the scanning was performed after wound healing.

Limited number of clinical trials have assessed the accuracy of intraoral digital impressions for complete-arch implant rehabilitations [16–19]. In an RCT, digital or conventional impressions were made for immediate “all-on-four” restorations. Radiographs were taken to verify marginal fit of the frameworks, and 12-month follow-up examinations were performed to evaluate peri-implant bone loss. The authors concluded that digital impressions could offer clinically satisfactory accuracy [16]. Similar conclusion was drawn by a more recent study with 24-month follow-up [18]. In a prospective study, digitalized conventional stone casts were served as reference data, and intraoral scanning demonstrated a mean deviation of 162 μm [20]. One of the major obstacles was that no suitable protocol was available to obtained reference data representing the ‘true’ implant positions from the patients’ oral cavity [15]. Radiographic and clinical examinations could not directly and sensitively represent the accuracy of impressions. The present study introduced an innovative study protocol that closely simulated the clinical situation, and meanwhile obtained the reference data by scanning the dogs’ mandibles using a lab scanner. The results could offer a reliable reference for the clinical application of digital impressions.

Generally, virtual models from intraoral scanning are created by overlapping of the images captured by an intraoral scanner. The process would inevitably introduce stitching errors. As reported in several literatures, the errors are more notable for fully edentulous arch due to the lack of stable feature points at the inter-implant regions [2, 15, 19, 22]. The same problems also presented in this study, especially when using SB. The smooth and reflective surface of the mucosa failed to provide enough reference points, which were critical to the images stitching process. What’s more, due to the limited amount of keratinized mucosa, the peri-implant mucosa was quite unstable. The mean linear and angular deviations of digital impressions using SB were 155.15 μm and 1.35 degrees in Dog 4. A possible explanation for the high deviations was that the lingual soft tissue of Dog 4 was severely swelling during the scanning, which was difficult to be stabilized. Additionally, it happened several times when scanning SB that the intraoral scanners could not distinguish one from another. As a result, one scan body or three scan bodies presented in the images instead of two separated scan bodies.

To overcome the difficulties, some techniques have been introduced recently. In an in vitro study, three modified scanning techniques, including application of glass beads or pressure-indicating paste on the mucosa surface and scan bodies splinted with dental floss, were investigated but showed no superiority on scanning accuracy compared to the control group with no modification [22]. Iturrate and coworkers introduced an auxiliary geometry piece to provide more characteristic reference point at the inter-implant regions concluded that the technique significantly improved the scanning accuracy [23]. In a clinical trial, digital impressions were made with the scan bodies splinted using wire and composite resin, and the definite restorations fabricated from digital impressions exhibited satisfactory clinical and radiological outcomes [18]. In this study, effort has been made to improve the scanning accuracy by introducing the scan bodies with extensional structure. The extensional structures were located at the inter-implant regions to make the scan bodies ‘splinted’ together. Reference points between two scan bodies were provided by the rigid structures instead of unstable mucosa, which could facilitate the stitching process and reduce stitching errors. Also, the extensional structure reduced the chances that the scanner confused the different scan bodies. Compared to the modified technique mentioned above, the application of SBE might be superior in terms of patients’ preference because no additional operation is needed in the patients’ oral cavity. The results indicated that the application of SBE significantly improved the trueness. Interestingly, the application of SBE markedly reduced both linear and angular deviations in Dog 4, suggesting that cases with unstable soft tissue might benefit more from SBE.

The accuracy of digital impressions was influenced by the fit between the scan body and the implant [33, 34]. Kim et al. reported that settling of the abutments increased with increasing tightening torques, and internal connected abutments developed much higher settling than external connected abutment [35]. Chia et al. found a mean horizontal discrepancy of 4–7 μm and vertical discrepancy of 11 μm with applied torque of 15 Ncm. Additionally, the study found that none of the scan bodies achieved perfect coaxiality with the implants [36]. The connection type of the scan bodies in this study was internal conical connection. There was no definite platform to serve as a vertical stop for correct positioning, which might introduce discrepancy. Therefore, the deviations between the test scans and the reference scans could partially come from positioning of the scan bodies, because the scan bodies were removed after the intraoral scanning and reconnected before obtaining the reference scans. However, the scan bodies in this study were made with titanium alloy, which might develop lower compressive deformation compared to polyether-ether-ketone (PEEK) scan bodies used in the aforementioned study. Also, a tightening torque of 10 Ncm was consistently applied to minimize the discrepancy. Thus, the deviations from the positioning were considered to be ignorable in this study.

The scanning was performed on unilateral mandibles instead of fully edentulous mandibles. However, the scanning procedure involved no tooth, but only the edentulous space. Considering the efforts to simulate a clinical situation of fully edentulous arch, one of the limitations is that the curve of dental arch was not present in this study. Additionally, the mandibles were fixed and the tongues were motionless because the dogs were under general anesthesia, which might facilitate the scanning procedure.

The clinical implication of the present study was that the innovative design of the extension structure on the scan body could be a solution to overcome the difficulty in digital impressions on fully edentulous arch. The results could serve as reliable reference particularly for the clinical situation of mandibular overdenture restorations with 2 implants. Further studies involving more implants and clinical trials are in need to support its clinical application.

## Conclusion

The application of SBE significantly improved the trueness of digital impressions in mandible with two implants compared to SB. No significant difference was found in terms of precision.

## Data Availability

The datasets used and/or analyzed during the current study are available from the corresponding author on reasonable request.

## Abbreviations

SB: Scan body without extensional structure
SBE: Scan body with extensional structure
CAD: Computer-aided design
STL: Standard Tessellation Language
RCT: Randomized controlled trial
PEEK: Polyether-ether-ketone

## References

[1] Joda T, Lenherr P, Dedem P, Kovaltschuk I, Bragger U, Zitzmann NU (2017). Time efficiency, difficulty, and operator's preference comparing digital and conventional implant impressions: a randomized controlled trial. Clin Oral Implants Res.

[2] Wulfman C, Naveau A, Rignon-Bret C (2020). Digital scanning for complete-arch implant-supported restorations: a systematic review. J Prosthet Dent.

[3] Kihara H, Hatakeyama W, Komine F, Takafuji K, Takahashi T, Yokota J (2020). Accuracy and practicality of intraoral scanner in dentistry: a literature review. J Prosthodont Res.

[4] Sawase T, Kuroshima S (2020). The current clinical relevancy of intraoral scanners in implant dentistry. Dent Mater J.

[5] Mangano F, Gandolfi A, Luongo G, Logozzo S (2017). Intraoral scanners in dentistry: a review of the current literature. BMC Oral Health.

[6] Joda T, Bragger U (2016). Patient-centered outcomes comparing digital and conventional implant impression procedures: a randomized crossover trial. Clin Oral Implants Res.

[7] Wismeijer D, Joda T, Flugge T, Fokas G, Tahmaseb A, Bechelli D (2018). Group 5 ITI Consensus Report: Digital technologies. Clin Oral Implants Res.

[8] Ahlholm P, Sipila K, Vallittu P, Jakonen M, Kotiranta U (2018). Digital versus conventional impressions in fixed prosthodontics: a review. J Prosthodont.

[9] Ender A, Mehl A (2013). Accuracy of complete-arch dental impressions: a new method of measuring trueness and precision. J Prosthet Dent.

[10] Papaspyridakos P, Gallucci GO, Chen CJ, Hanssen S, Naert I, Vandenberghe B (2016). Digital versus conventional implant impressions for edentulous patients: accuracy outcomes. Clin Oral Implants Res.

[11] Amin S, Weber HP, Finkelman M, El Rafie K, Kudara Y, Papaspyridakos P (2017). Digital vs. conventional full-arch implant impressions: a comparative study. Clin Oral Implants Res.

[12] Alikhasi M, Siadat H, Nasirpour A, Hasanzade M (2018). Three-dimensional accuracy of digital impression versus conventional method: effect of implant angulation and connection type. Int J Dent.

[13] Menini M, Setti P, Pera F, Pera P, Pesce P (2018). Accuracy of multi-unit implant impression: traditional techniques versus a digital procedure. Clin Oral Investig.

[14] Di Fiore A, Meneghello R, Graiff L, Savio G, Vigolo P, Monaco C (2019). Full arch digital scanning systems performances for implant-supported fixed dental prostheses: a comparative study of 8 intraoral scanners. J Prosthodont Res.

[15] Flugge T, van der Meer WJ, Gonzalez BG, Vach K, Wismeijer D, Wang P (2018). The accuracy of different dental impression techniques for implant-supported dental prostheses: a systematic review and meta-analysis. Clin Oral Implants Res.

[16] Gherlone E, Cappare P, Vinci R, Ferrini F, Gastaldi G, Crespi R (2016). Conventional versus digital impressions for "All-on-Four" restorations. Int J Oral Maxillofac Implants.

[17] Gherlone EF, Ferrini F, Crespi R, Gastaldi G, Cappare P (2015). Digital impressions for fabrication of definitive "all-on-four" restorations. Implant Dent.

[18] Cappare P, Sannino G, Minoli M, Montemezzi P, Ferrini F (2019). Conventional versus digital impressions for full arch screw-retained maxillary rehabilitations: a randomized clinical trial. Int J Environ Res Public Health.

[19] Andriessen FS, Rijkens DR, van der Meer WJ, Wismeijer DW (2014). Applicability and accuracy of an intraoral scanner for scanning multiple implants in edentulous mandibles: a pilot study. J Prosthet Dent.

[20] Chochlidakis K, Papaspyridakos P, Tsigarida A, Romeo D, Chen Y W, Natto Z, et al. Digital Versus Conventional Full-Arch Implant Impressions: A Prospective Study on 16 Edentulous Maxillae. J Prosthodont. 2020. 10.1111/jopr.13162 32166793

[21] Nedelcu R, Olsson P, Nystrom I, Ryden J, Thor A (2018). Accuracy and precision of 3 intraoral scanners and accuracy of conventional impressions: a novel in vivo analysis method. J Dent.

[22] Mizumoto RM, Yilmaz B, McGlumphy EA Jr., Seidt J, Johnston WM. Accuracy of different digital scanning techniques and scan bodies for complete-arch implant-supported prostheses. J Prosthet Dent. 2020;123(1):96–104 10.1016/j.prosdent.2019.01.003 31040026

[23] Iturrate M, Eguiraun H, Solaberrieta E (2019). Accuracy of digital impressions for implant-supported complete-arch prosthesis, using an auxiliary geometry part—an in vitro study. Clin Oral Implants Res.

[24] Iturrate M, Minguez R, Pradies G, Solaberrieta E (2019). Obtaining reliable intraoral digital scans for an implant-supported complete-arch prosthesis: a dental technique. J Prosthet Dent.

[25] Huang R, Liu Y, Huang B, Zhang C, Chen Z, Li Z (2020). Improved scanning accuracy with newly designed scan bodies: an in vitro study comparing digital versus conventional impression techniques for complete-arch implant rehabilitation. Clin Oral Implants Res.

[26] Jemt T, Lie A (1995). Accuracy of implant-supported prostheses in the edentulous jaw: analysis of precision of fit between cast gold-alloy frameworks and master casts by means of a three-dimensional photogrammetric technique. Clin Oral Implants Res.

[27] Tan MY, Yee SHX, Wong KM, Tan YH, Tan KBC (2019). Comparison of three-dimensional accuracy of digital and conventional implant impressions: effect of interimplant distance in an edentulous arch. Int J Oral Maxillofac Implants.

[28] Kim KR, Seo KY, Kim S (2019). Conventional open-tray impression versus intraoral digital scan for implant-level complete-arch impression. J Prosthet Dent.

[29] Vandeweghe S, Vervack V, Dierens M, De Bruyn H (2017). Accuracy of digital impressions of multiple dental implants: an in vitro study. Clin Oral Implants Res.

[30] Imburgia M, Logozzo S, Hauschild U, Veronesi G, Mangano C, Mangano FG (2017). Accuracy of four intraoral scanners in oral implantology: a comparative in vitro study. BMC Oral Health.

[31] Mangano FG, Hauschild U, Veronesi G, Imburgia M, Mangano C, Admakin O (2019). Trueness and precision of 5 intraoral scanners in the impressions of single and multiple implants: a comparative in vitro study. BMC Oral Health.

[32] Arcuri L, Pozzi A, Lio F, Rompen E, Zechner W, Nardi A (2020). Influence of implant scanbody material, position and operator on the accuracy of digital impression for complete-arch: a randomized in vitro trial. J Prosthodont Res.

[33] Mizumoto RM, Yilmaz B (2018). Intraoral scan bodies in implant dentistry: a systematic review. J Prosthet Dent.

[34] Pan Y, Tam JMY, Tsoi JKH, Lam WYH, Pow EHN (2020). Reproducibility of laboratory scanning of multiple implants in complete edentulous arch: effect of scan bodies. J Dent.

[35] Kim KS, Lim YJ, Kim MJ, Kwon HB, Yang JH, Lee JB (2011). Variation in the total lengths of abutment/implant assemblies generated with a function of applied tightening torque in external and internal implant-abutment connection. Clin Oral Implants Res.

[36] Chia VA, Esguerra RJ, Teoh KH, Teo JW, Wong KM, Tan KB (2017). In vitro three-dimensional accuracy of digital implant impressions: the effect of implant angulation. Int J Oral Maxillofac Implants.

